# Supplementary material for: Tapping into non-English-language science for the conservation of global biodiversity
Source: PLoS Biol. 2021 Oct 7;19(10):e3001296. doi: 10.1371/journal.pbio.3001296 (PMC8496809; doi:10.1371/journal.pbio.3001296)
Supplement: S1 Table — Significant results are shown in bold. BA, Before–After; BACI, Before–After–Control–Impact; CI, Control–Impact; RCT, Randomised Controlled Trial. (DOCX) [file pbio.3001296.s001.docx]

**Table S1.** Results of a cumulative link model aimed at testing the association between ordered study designs (with Randomised Controlled Trial as the least biased design, followed by Before-After-Control-Impact, Control-Impact, Before-After, and After) in each study as the response variable, and languages (16 non-English languages and “English – official” (English-language studies conducted in countries where English is an official language), compared to “English – others” (English-language studies conducted in the other countries) as the reference category) and taxa (birds, mammals, and others, compared to amphibians as the reference category) as the explanatory variables. Significant results are shown in bold.

| Coefficients | Estimate | Standard error | *z* | *p* |
| --- | --- | --- | --- | --- |
| **Arabic** | **-3.14** | **1.08** | **-2.92** | **0.0035** |
| **English – official** | **0.18** | **0.055** | **3.32** | **0.00091** |
| **French** | **-0.60** | **0.20** | **-2.93** | **0.0034** |
| **German** | **-1.34** | **0.13** | **-10.47** | **1.16 × 10^-25^** |
| **Hungarian** | **-0.57** | **0.24** | **-2.39** | **0.017** |
| **Italian** | **-2.42** | **0.49** | **-4.91** | **9.15 × 10^-7^** |
| **Japanese** | **-0.91** | **0.11** | **-8.16** | **3.31 × 10^-16^** |
| **Korean** | **-2.56** | **0.37** | **-6.85** | **7.44 × 10^-12^** |
| Persian | 0.067 | 0.53 | 0.13 | 0.90 |
| **Polish** | **-0.99** | **0.22** | **-4.44** | **8.97 × 10^-6^** |
| Portuguese | 0.49 | 0.26 | 1.90 | 0.057 |
| **Russian** | **-1.64** | **0.24** | **-6.84** | **7.67 × 10^-12^** |
| **Simplified Chinese** | **-0.67** | **0.18** | **-3.79** | **0.00015** |
| Spanish | -0.33 | 0.17 | -1.87 | 0.061 |
| Traditional Chinese | 0.16 | 0.38 | 0.42 | 0.67 |
| Turkish | 0.69 | 1.08 | 0.64 | 0.52 |
| Ukrainian | 0.52 | 1.10 | 0.47 | 0.64 |
| Taxa – Birds | -0.13 | 0.11 | -1.22 | 0.22 |
| Taxa – Mammals | -0.15 | 0.11 | -1.35 | 0.18 |
| **Taxa – others** | **0.83** | **0.10** | **7.95** | **1.83 × 10^-15^** |
