# Supplementary material for: Tapping into non-English-language science for the conservation of global biodiversity
Source: PLoS Biol. 2021 Oct 7;19(10):e3001296. doi: 10.1371/journal.pbio.3001296 (PMC8496809; doi:10.1371/journal.pbio.3001296)
Supplement: S2 Table — (DOCX) [file pbio.3001296.s002.docx]

**Table S2**. List of those involved in searches and their roles for each language covered in this study.

| **Language** | **Name** | **Journal listing** | **Searches** | **Data coding** | **Validation** |
| --- | --- | --- | --- | --- | --- |
| Arabic | Perla Farhat | 1 | 1 | 1 | 0 |
| Arabic | Magda Bou Dagher Kharrat | 1 | 0 | 0 | 0 |
| French | Ingrid Pollet | 1 | 1 | 1 | 0 |
| French | Marie-Morgane Rouyer | 1 | 1 | 1 | 0 |
| French | Ana Reboredo Segovia | 0 | 1 | 1 | 0 |
| German | Dominik Schwab | 1 | 1 | 1 | 0 |
| German | Kerstin Jantke | 1 | 1 | 1 | 0 |
| German | Isabel Mangold | 1 | 1 | 1 | 0 |
| German | Horst Korn | 1 | 0 | 0 | 0 |
| German | Richard Schuter | 0 | 0 | 1 | 0 |
| German | Matthias-Claudio Loretto | 0 | 0 | 1 | 0 |
| Hungarian | Flóra Vajna | 1 | 1 | 1 | 0 |
| Hungarian | András Báldi | 1 | 0 | 0 | 0 |
| Italian | Sandro Bertolino | 1 | 1 | 1 | 0 |
| Italian | Valentina Marconi | 0 | 1 | 1 | 0 |
| Japanese | Ko Konno | 1 | 1 | 1 | 0 |
| Japanese | Munemitsu Akasaka | 1 | 0 | 0 | 0 |
| Japanese | Yushin Shinoda | 1 | 1 | 1 | 0 |
| Japanese, simplified Chinese | Tatsuya Amano | 1 | 1 | 1 | 1 |
| Japanese | Kensuke Kito | 0 | 0 | 1 | 0 |
| Korean | Hae-Min Seo | 1 | 1 | 1 | 0 |
| Korean | Chang-Yong Choi | 1 | 0 | 0 | 0 |
| Persian | Elham Nourani | 1 | 1 | 1 | 0 |
| Polish | Joanna Kajzer-Bonk | 1 | 1 | 1 | 0 |
| Polish | Pawel Waryszak | 0 | 1 | 1 | 0 |
| Portuguese | Ana Cláudia Piovezan Borges | 1 | 1 | 1 | 0 |
| Portuguese | Rafael D. Zenni | 0 | 1 | 1 | 0 |
| Portuguese | Danielle Ramos | 1 | 1 | 1 | 0 |
| Portuguese | Jose Manuel Ochoa Quintero | 1 | 0 | 0 | 0 |
| Portuguese | Juan Pablo Narváez-Gómez | 0 | 1 | 1 | 0 |
| Portuguese | Luis Gustavo de Oliveira | 0 | 0 | 1 | 0 |
| Portuguese and Spanish | Ricardo Rocha | 1 | 1 | 1 | 1 |
| Russian | Igor Khorozyan | 1 | 1 | 1 | 0 |
| Russian | Svetlana Vozykova | 0 | 1 | 1 | 0 |
| Simplified Chinese | Yifan Liu | 1 | 1 | 1 | 0 |
| Simplified Chinese | Min Chen | 1 | 1 | 1 | 0 |
| Simplified Chinese | Wenjun Zhou | 1 | 1 | 1 | 0 |
| Simplified Chinese | Yang Liu | 1 | 1 | 1 | 0 |
| Simplified Chinese | Rachel Oh | 0 | 0 | 1 | 0 |
| Spanish | Jose Valdebenito Chavez | 1 | 1 | 1 | 0 |
| Spanish | Nataly Hidalgo Aranzamendi | 1 | 1 | 1 | 0 |
| Spanish | Nayelli Rivera | 0 | 1 | 1 | 0 |
| Spanish | Veronica Zamora-Gutierrez | 1 | 0 | 0 | 0 |
| Spanish | Pablo Jose Negret | 0 | 0 | 1 | 0 |
| Traditional Chinese | Ming-shan Tsai | 1 | 1 | 1 | 0 |
| Traditional Chinese | Shan-dar Tao | 1 | 1 | 1 | 0 |
| Traditional Chinese | Da-Li Lin | 0 | 1 | 1 | 0 |
| Turkish | M. Çisel Kemahlı Aytekin | 1 | 1 | 1 | 0 |
| Turkish | Çağan Hakkı Şekercioğlu | 1 | 0 | 0 | 0 |
| Ukrainian | Marina Golivets | 1 | 1 | 1 | 0 |
| Arabic, French, German, Italian, Korean, Polish, Spanish, Russian, Ukrainian | Kate Willott | 0 | 0 | 0 | 1 |
| Traditional Chinese, French | William Morgan | 0 | 0 | 0 | 1 |
| French, Portuguese | Philip Martin | 0 | 0 | 0 | 1 |
| French, German, Hungarian | Katherine Sainsbury | 0 | 0 | 0 | 1 |
| German, Simplified Chinese | Elizabeth Tyler | 0 | 0 | 0 | 1 |
| French, Hungarian, Spanish | Andrew Bladon | 0 | 0 | 0 | 1 |
| Japanese, Korean, Persian, Polish, Simplified Chinese | Rebecca Smith | 0 | 0 | 0 | 1 |
| Japanese, Simplified Chinese | Nancy Ockendon | 0 | 0 | 0 | 1 |
| Japanese | Gorm Shackelford | 0 | 0 | 0 | 1 |
| Japanese, Simplified Chinese | Nick Littlewood | 0 | 0 | 0 | 1 |
| Japanese, Polish, Simplified Chinese, Turkish | Silviu Petrovan | 0 | 0 | 0 | 1 |
| Spanish | Anna Berthinussen | 0 | 0 | 0 | 1 |
