# Supplementary material for: Tapping into non-English-language science for the conservation of global biodiversity
Source: PLoS Biol. 2021 Oct 7;19(10):e3001296. doi: 10.1371/journal.pbio.3001296 (PMC8496809; doi:10.1371/journal.pbio.3001296)
Supplement: S1 Fig — Coloured dots connected with a line represent all journals screened for each language, in decreasing order of % eligible studies; the journal with the highest % eligible studies is shown on the far left, while the journal with the lowest % eligible studies is on the far right. This figure was created using S1 Data and Code 5. (DOCX) [file pbio.3001296.s003.docx]

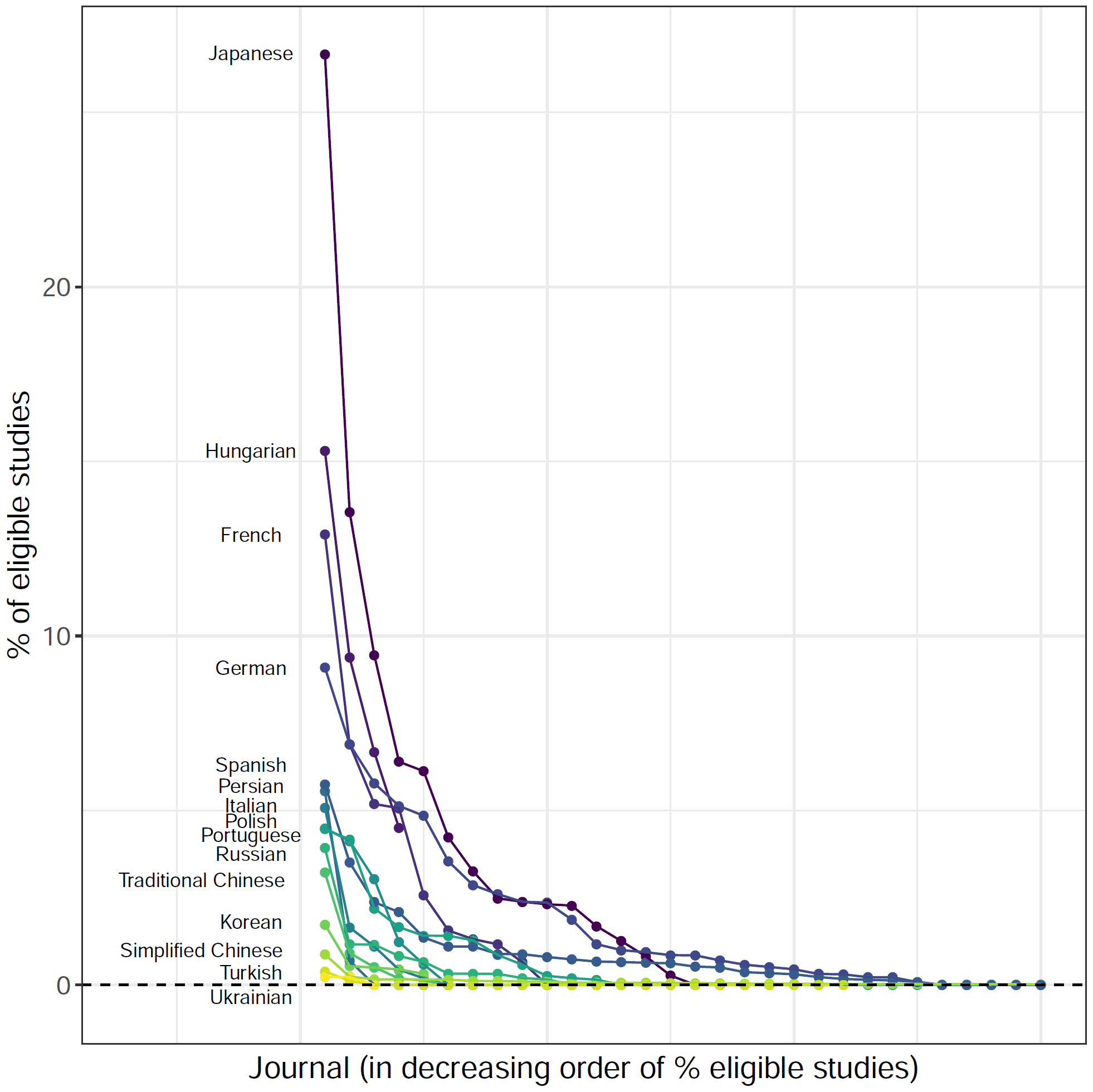


**S1 Fig.** The proportion (%) of eligible studies testing the effectiveness of conservation interventions in each journal in 16 non-English languages. Coloured dots connected with a line represent all journals screened for each language, in decreasing order of % eligible studies; the journal with the highest % eligible studies is shown on the far left, while the journal with the lowest % eligible studies is on the far right. This figure was created using S1 Data and Code 5.
