# Supplementary material for: Tapping into non-English-language science for the conservation of global biodiversity
Source: PLoS Biol. 2021 Oct 7;19(10):e3001296. doi: 10.1371/journal.pbio.3001296 (PMC8496809; doi:10.1371/journal.pbio.3001296)
Supplement: S2 Fig — This figure was created using S3 and S4 Data with Code 3. Map produced from the Natural Earth dataset (v.4.1.0) at 1:50 m scale (https://www.naturalearthdata.com/downloads/50m-cultural-vectors/). (DOCX) [file pbio.3001296.s004.docx]

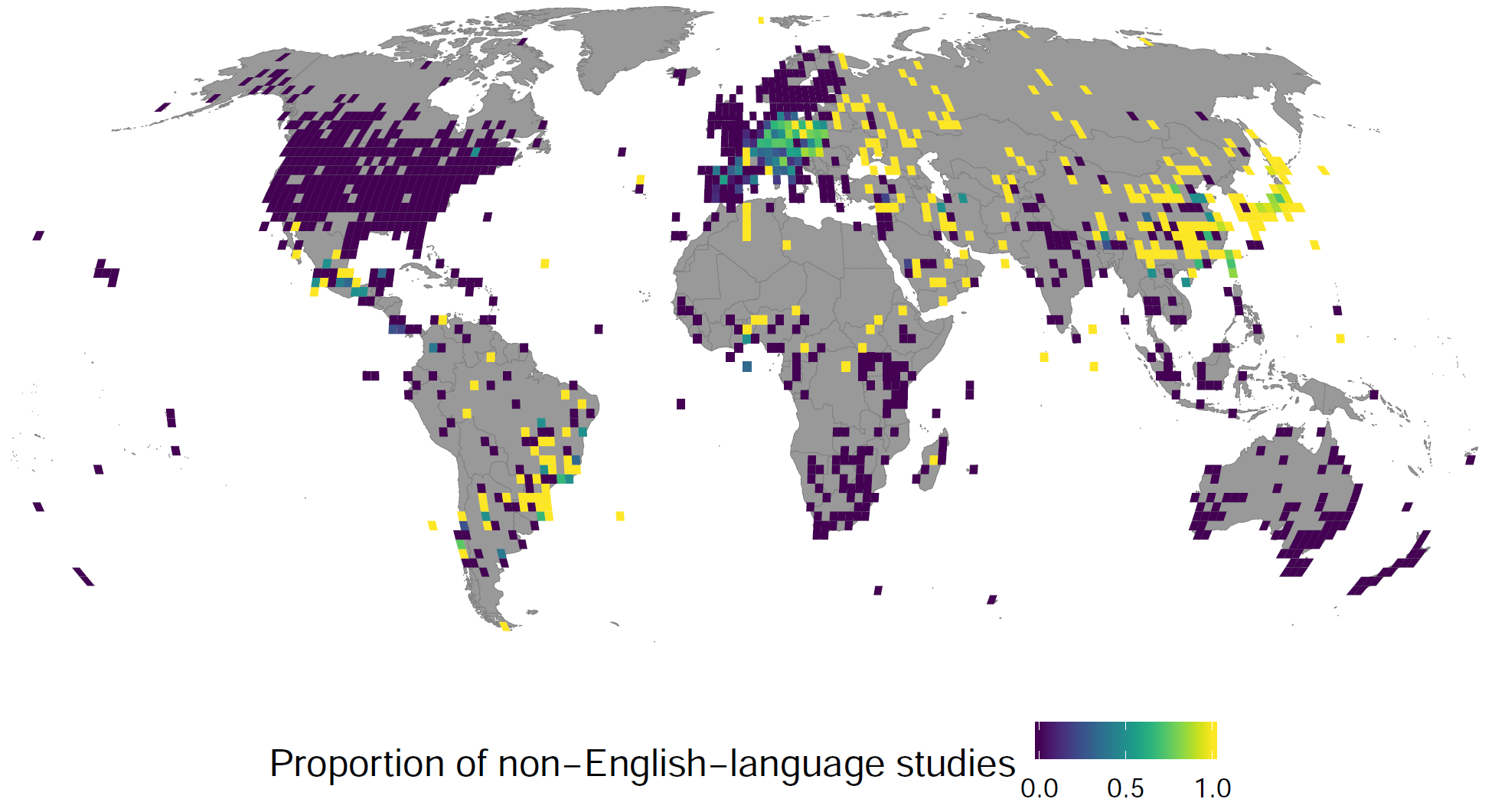


**S2 Fig.** The proportion of non-English-language studies (all 16 languages combined) to all studies (i.e., non-English and English-language studies combined) testing the effectiveness of conservation interventions within each 2° × 2° grid cell. This figure was created using S3 and S4 Data with Code 3. Map produced from the Natural Earth dataset (v.4.1.0) at 1:50m scale (https://www.naturalearthdata.com/downloads/50m-cultural-vectors/).
