# Supplementary material for: Tapping into non-English-language science for the conservation of global biodiversity
Source: PLoS Biol. 2021 Oct 7;19(10):e3001296. doi: 10.1371/journal.pbio.3001296 (PMC8496809; doi:10.1371/journal.pbio.3001296)
Supplement: S4 Fig — Non-English-language studies were found in 75 grid cells, 59 of which were without any English-language studies (grid cells in black). The inset is a hexbin chart showing a significantly negative relationship between the number of English-language studies (No. English studies) and the number of non-English-language studies (No. non-English studies) within each grid cell. Brighter colours indicate more grid cells in each hexagon. This figure was created using S3 and S4 Data with Code 6. Map produced from the Natural Earth dataset (v.4.1.0) at 1:5 0m scale (https://www.naturalearthdata.com/downloads/50m-cultural-vectors/). (DOCX) [file pbio.3001296.s006.docx]

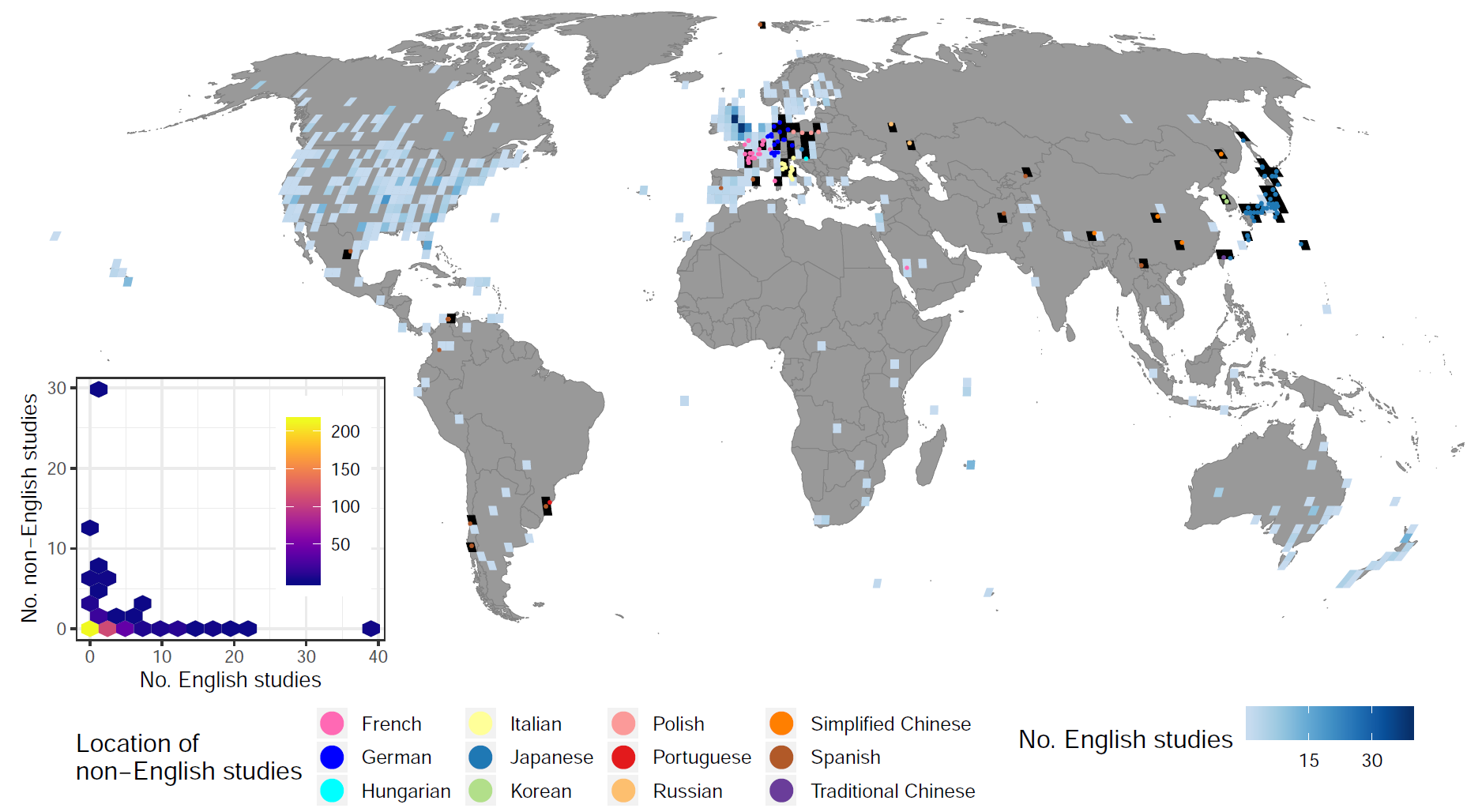


**S4 Fig.** The location of 182 non-English-language studies testing the effectiveness of conservation interventions for bird species (published in 2011 or earlier), compared to the number of English-language studies on birds within each 2° × 2° grid cell (373 grid cells in total). Non-English-language studies were found in 75 grid cells, 59 of which were without any English language studies (grid cells in black). The inset is a hexbin chart showing a significantly negative relationship between the number of English-language studies (No. English studies) and the number of non-English-language studies (No. non-English studies) within each grid cell. Brighter colours indicate more grid cells in each hexagon. This figure was created using S3 and S4 Data with Code 6. Map produced from the Natural Earth dataset (v.4.1.0) at 1:50m scale (https://www.naturalearthdata.com/downloads/50m-cultural-vectors/).
