# Supplementary material for: Tapping into non-English-language science for the conservation of global biodiversity
Source: PLoS Biol. 2021 Oct 7;19(10):e3001296. doi: 10.1371/journal.pbio.3001296 (PMC8496809; doi:10.1371/journal.pbio.3001296)
Supplement: S6 Fig — Note that a threatened mammal species with 38 English-language studies is not shown as an outlier. The insets are hexbin charts showing the relationship between the number of English-language studies (No. English studies) and the number of non-English-language studies (No. non-English studies) for each threatened species. Species classified as threatened (Critically Endangered, Endangered, or Vulnerable) based on IUCN. Brighter colours indicate more species in each hexagon. Only studies published in 2012 or earlier for amphibians, 2011 or earlier for birds, and 2018 or earlier for mammals were used in this figure. This figure was created using S3 and S5 Data with Code 4 and IUCN species lists available at: https://www.iucnredlist.org/resources/spatial-data-download. (DOCX) [file pbio.3001296.s008.docx]

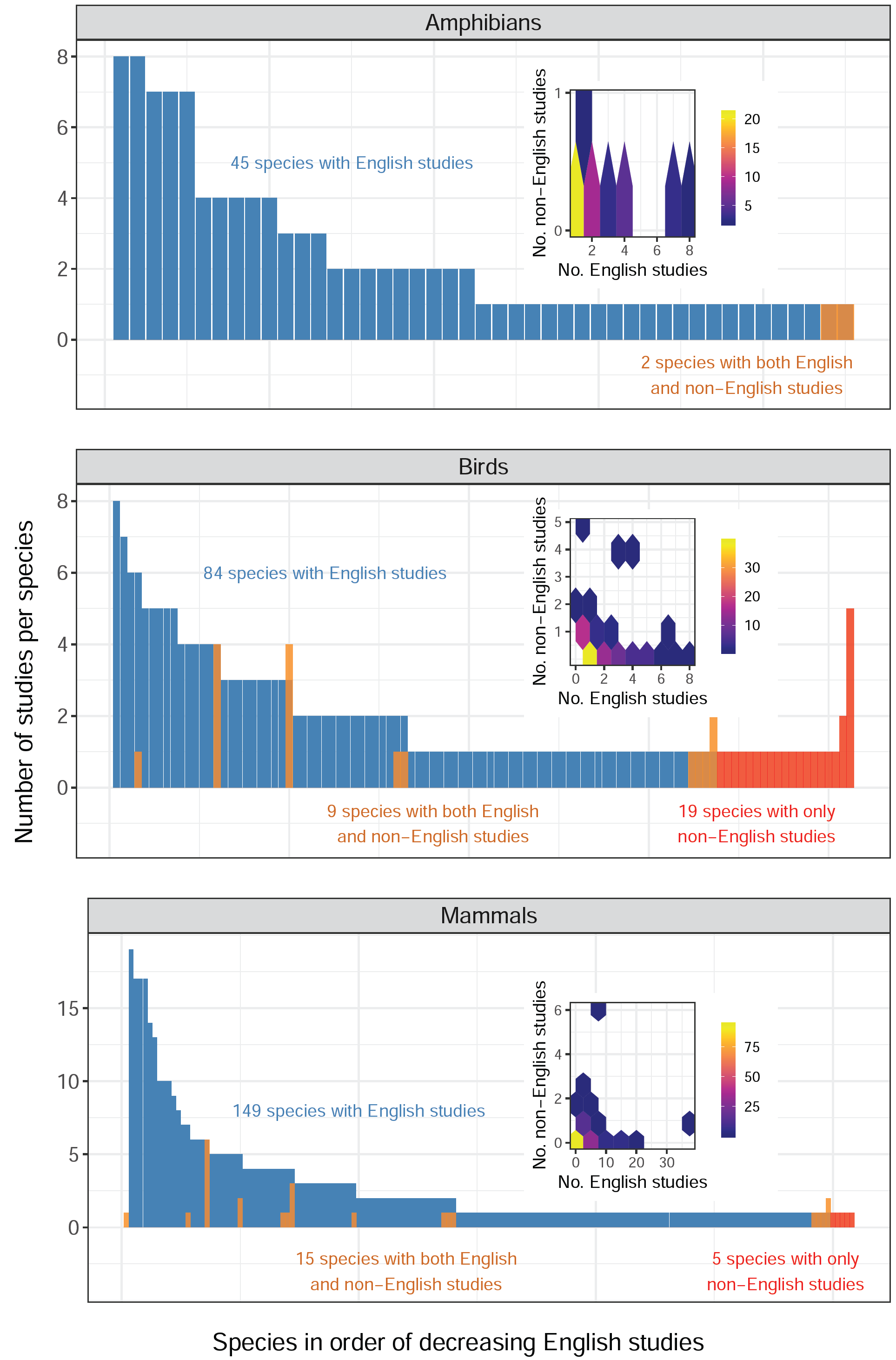


**S6 Fig.** The distribution of the number of English-language studies for each threatened amphibian, bird and mammal species (blue), with species ranked on the x axis in order of decreasing number of English-language studies, and the number of non-English-language studies per species for those threatened species studied by both English- and non-English-language studies (orange), and those studied only by non-English-language studies (red). Note that a threatened mammal species with 38 English-language studies is not shown as an outlier. The insets are hexbin charts showing the relationship between the number of English-language studies (No. English studies) and the number of non-English-language studies (No. non-English studies) for each threatened species. Species classified as threatened (Critically Endangered, Endangered or Vulnerable) based on IUCN. Brighter colours indicate more species in each hexagon. Only studies published in 2012 or earlier for amphibians, 2011 or earlier for birds and 2018 or earlier for mammals were used in this figure. This figure was created using S3 and S5 Data with Code 4 and IUCN species lists available at: https://www.iucnredlist.org/resources/spatial-data-download.
