# Supplementary material for: Tapping into non-English-language science for the conservation of global biodiversity
Source: PLoS Biol. 2021 Oct 7;19(10):e3001296. doi: 10.1371/journal.pbio.3001296 (PMC8496809; doi:10.1371/journal.pbio.3001296)
Supplement: S1 Alternative Language Abstract — (PDF) [file pbio.3001296.s014.pdf]

Puiser dans les sciences non-anglophones pour la conservation de la biodiversité mondiale

Résumé

L'hypothèse largement répandue selon laquelle toute information scientifique importante serait disponible en anglais sous-tend la sous-utilisation de la science non-anglophone dans toutes les disciplines. La science non-anglophone peut néanmoins apporter des informations scientifiques uniques et précieuses, en particulier dans les disciplines où les données sont disparates et pour les problématiques émergentes où la synthèse des connaissances factuelles existantes est un défi urgent. Cependant, cette contribution de la science non-anglophone aux communautés scientifiques et à l'application de la science est rarement quantifiée. Ici, nous montrons que les études non-anglophones fournissent des preuves cruciales pouvant étayer la conservation mondiale de la biodiversité. En passant au crible 419 679 articles évalués par un comité de lecture dans 16 langues, nous avons identifié 1 234 études non-anglophones démontrant l'efficacité d’interventions de conservation de la biodiversité, contre 4 412 études anglophones identifiées avec les mêmes critères. Les études non-anglophones applicables sont publiées à un rythme croissant dans six des douze langues pour lesquelles le nombre d'études applicables était suffisant. La prise en compte des études non-anglophones peut étendre la couverture géographique (c'est-à-dire le nombre de cellules d’une grille de 2° × 2° comprenant des études applicables) des preuves apportées par les études anglophones de 12 à 25 %, en particulier dans les régions riches en biodiversité, et la couverture taxonomique (c'est-à-dire le nombre d'espèces inclus dans les études applicables) de 5 à 32 %, bien qu'elles aient tendance à être basées sur des protocoles d’étude moins solides. Nos résultats démontrent que la synthèse des études non-anglophones est essentielle pour surmonter le manque généralisé de preuves locales et contextuelles, et pour faciliter la prise en compte d’interventions ayant fait leurs preuves dans la conservation de la biodiversité au niveau mondial. Nous encourageons l’ensemble des disciplines à réévaluer rigoureusement le potentiel inexploité de la science non-anglophone pour éclairer les décisions visant à relever d'autres défis mondiaux.

Translated by Marie-Morgane Rouyer
