# Supplementary material for: Tapping into non-English-language science for the conservation of global biodiversity
Source: PLoS Biol. 2021 Oct 7;19(10):e3001296. doi: 10.1371/journal.pbio.3001296 (PMC8496809; doi:10.1371/journal.pbio.3001296)
Supplement: S2 Alternative Language Abstract — (PDF) [file pbio.3001296.s015.pdf]

Erschließung nicht-englischsprachiger Wissenschaftsbeiträge zur Erhaltung der globalen Biodiversität

Abstract

Die weit verbreitete Annahme, dass alle wichtigen wissenschaftlichen Informationen auf Englisch verfügbar wären, basiert auf unzureichender Nutzung nicht-englischsprachiger Wissenschaftsbeiträge in allen Disziplinen. Es wird jedoch vermutet, dass auch nicht-englischsprachige Wissenschaftsbeiträge einzigartige und wertvolle wissenschaftliche Informationen liefern. Dies gilt besonders für Bereiche mit lückenhafter Faktenlage oder für aufkommende Probleme, bei denen die Suche nach Fakten eine dringende Herausforderung darstellt. Es wird nur selten quantifiziert inwiefern nicht-englischsprachige Wissenschaftsbeiträge die Wissenschaftsgemeinschaft sowie die Umsetzung in der Wissenschaft beeinflussen. Wir zeigen, dass nicht-englischsprachige Studien entscheidende Informationen für den globalen Artenschutzes liefern. Durch das Sichten von 419.679 Peer-Review-Artikeln in 16 Sprachen identifizierten wir 1.234 nicht-englischsprachige Studien, die wichtige Informationen hinsichtlich der Wirksamkeit von Maßnahmen zum Schutz der Biodiversität liefern. Im Vergleich wurden mit denselben Kriterien 4.412 englischsprachige Studien identifiziert. In sechs von 12 Sprachen mit ausreichend relevanten Studien steigt die Anzahl relevanter nicht englischsprachiger Publikationen. Wenn neben den englischsprachigen Studien auch die nicht englischsprachigen Studien einbezogen werden, kann sowohl die geografische Abdeckung (die Anzahl der 2° × 2°-Rasterzellen mit relevanten Studien) um 12-25% (besonders in artenreichen Regionen), als auch die taxonomische Abdeckung (d.h.: die Anzahl der Arten, die von den relevanten Studien abgedeckt werden) um 5–32 % erweitert werden, obwohl nicht-englischsprachige Beiträge tendenziell auf weniger robusten Studienaufbau basieren. Unsere Ergebnisse zeigen, dass ein Einbinden nicht-englischsprachiger Beiträge ein wichtiger Schritt ist den weit verbreiteten Mangel an lokalen, kontextabhängigen Fakten zu überwinden und den evidenzbasierten Naturschutz weltweit zu verbessern. Wir rufen auch andere Disziplinen auf, das ungenutzte Potenzial der nicht-englischsprachigen Wissenschaftsbeiträge zur Bewältigung anderer globaler Herausforderungen im Detail neu zu bewerten.

Translated by Matthias Loretto und Richard Schuster
