# Supplementary material for: Tapping into non-English-language science for the conservation of global biodiversity
Source: PLoS Biol. 2021 Oct 7;19(10):e3001296. doi: 10.1371/journal.pbio.3001296 (PMC8496809; doi:10.1371/journal.pbio.3001296)
Supplement: S3 Alternative Language Abstract — (PDF) [file pbio.3001296.s016.pdf]

A nem angol nyelvű szakirodalom bevonása a globális biodiverzitás megőrzése érdekében

Összefoglaló

Általános vélekedés szerint minden fontos tudományos információnak elérhetőnek kell lennie angolul is, amit hátráltat a nem angol nyelvű tudományos munkák használatának elhanyagolása. Pedig a nem angol nyelvű tudományos vizsgálatok egyedi és értékes tudományos tartalommal bírnak, különösen azon tudományterületeken, ahol a tudományos ismeretek hiányosak és ahol a felmerülő problémákra sürgősen adandó a válasz. Ez idáig a nem angol nyelvű tudományos munkák bevonása a tudományos közösségek munkájába és alkalmazásuk ritkán történt meg. Ebben a tanulmányban bemutatjuk, hogy a nem angol nyelvű tudományos közlemények alapvető bizonyítékokkal szolgálnak a globális biodiverzitás megőrzése érdekében. 16 nyelven íródott 419.679 szakcikk átnézése során azonosítottunk 1.234 olyan, nem angol nyelvű tanulmányt, amely bizonyítékkal szolgál a biológiai sokféleség megőrzése érdekében tett valamilyen beavatkozás hatékonyságáról; ezeket összehasonlítottunk 4.412 angol nyelvű közleménnyel, amelyeket azonos kritériumok alapján válogattunk ki. A releváns, nem angol nyelvű szakcikkek közzétételének üteme egyre növekszik 6 nyelv esetében, abból a 12 nyelvből ahol elegendő releváns tanulmány volt elérhető. A nem angol nyelvű vizsgálatok figyelembevételével 12-25%-kal növelhető a tudományos ismeretek földrajzi lefedettsége (azaz, a releváns tanulmányok száma 2° × 2° rácscellákban), főként a biológiailag diverz régiókban; és 5-32%-kal a taxonómiai lefedettség (azaz, a releváns közleményekben megjelent fajok száma), habár ezek a vizsgálatok kevésbé robusztus vizsgálati elrendezésen alapulnak. Eredményeink azt mutatják, hogy a nem angol nyelven íródott tanulmányok kulcsfontosságúak a helyi, kontextusfüggő ismeretek hiányának leküzdésében és a globális biológiai sokféleség megőrzésének elősegítésében. Arra buzdítjuk a különböző tudományágak művelőit, hogy értékeljék újra a nem angol nyelvű tudományos szakirodalom használatának eddig ki nem aknázott lehetőségeit, mikor globális kihívások esetében döntéseket hoznak és kezelik azokat.

Translated by Flóra Vajna and András Báldi
