# Supplementary material for: Tapping into non-English-language science for the conservation of global biodiversity
Source: PLoS Biol. 2021 Oct 7;19(10):e3001296. doi: 10.1371/journal.pbio.3001296 (PMC8496809; doi:10.1371/journal.pbio.3001296)
Supplement: S4 Alternative Language Abstract — (PDF) [file pbio.3001296.s017.pdf]

Attingere alla scienza non in lingua inglese per la conservazione della biodiversità globale

Riassunto

L'assunto che qualsiasi informazione scientifica importante sia disponibile in inglese è alla base del sottoutilizzo di quanto pubblicato non in lingua inglese in tutte le discipline. Tuttavia, ci si aspetta che la scienza non in lingua inglese possa aggiungere informazioni scientifiche uniche e preziose, specialmente nelle discipline dove le informazioni disponibili sono frammentarie e per temi emergenti per cui la sintesi delle evidenze disponibili è una sfida urgente. Ciò nonostante, il contributo della scienza non in lingua inglese al progresso della scienza in tempi recenti è raramente quantificato. Qui mostriamo come gli studi non in lingua inglese forniscano evidenze fondamentali per supportare la conservazione della biodiversità globale. Attraverso lo screening di 419.679 articoli sottoposti a revisione paritaria in 16 lingue, abbiamo identificato 1.234 studi non in lingua inglese che forniscono prove sull'efficacia degli interventi di conservazione della biodiversità, rispetto a 4.412 studi in lingua inglese identificati con gli stessi criteri. Studi rilevanti non in lingua inglese vengono pubblicati a un ritmo crescente in sei delle 12 lingue considerate, per cui era disponibile un numero sufficiente di studi rilevanti. Incorporare studi non in lingua inglese che riportano evidenze sull'efficacia degli interventi di conservazione, può espandere del 12-25% la copertura geografica di tali studi (cioè il numero di celle della griglia 2° × 2° con studi pertinenti) ottenuta considerando solo studi in lingua inglese, questo specialmente nelle regioni con biodiversità elevata. Allo stesso modo, la copertura tassonomica (cioè il numero di specie coperte dagli studi con evidenze utili alla conservazione) aumenta del 5-32%. D'altro canto, gli studi in lingua non inglese tendono a basarsi su disegni di studio meno robusti. I nostri risultati mostrano come includere nei lavori di sintesi studi non in lingua inglese possa aiutare a superare la diffusa mancanza di evidenze locali e dipendenti dal contesto e facilitare la conservazione basata sulle evidenze a livello globale. Infine, auspichiamo studi analoghi in altre discipline per poter valutare in maniera rigorosa il potenziale non sfruttato della scienza non in lingua inglese nell'informare le decisioni necessarie per affrontare sfide globali.

Translated by Sandro Bertolino
