# Supplementary material for: Tapping into non-English-language science for the conservation of global biodiversity
Source: PLoS Biol. 2021 Oct 7;19(10):e3001296. doi: 10.1371/journal.pbio.3001296 (PMC8496809; doi:10.1371/journal.pbio.3001296)
Supplement: S5 Alternative Language Abstract — (PDF) [file pbio.3001296.s018.pdf]

全世界の生物多様性保全のために英語以外の言語での科学を活用する

摘要

現代では重要な科学的情報は全て英語で入手できるという前提のもと、分野を問わず英語以外の言語で書かれた科学的知識は十分に活用されていない。しかし、特に科学的エビデンスが乏しい分野や、得られる全てのエビデンスを統合することが喫緊の課題であるような緊急性の高い問題については、英語以外の言語で発表されている科学が、固有且つ価値のある科学的情報をもたらすことが期待される。しかしながら、このような英語以外の言語で書かれた科学的知識が科学コミュニティや科学の応用に対してどのように貢献するのかは、ほとんど定量化されていない。本研究では、英語以外の言語で発表されている研究が、世界の生物多様性保全のために重要な科学的エビデンスを提供していることを明らかにした。16 言語で発表された 419,679 件の査読付き論文を精査した結果、生物多様性の保全対策の有効性に関するエビデンスについて、英語以外の言語で発表されている研究を 1,234 件特定した。同じ基準でこれまで特定された英語で発表されている研究は 4,412 件であった。十分な数の関連研究があった 12 言語のうち 6 言語では、関連する非英語研究の発表が年毎に増加していた。これらの英語以外の言語で発表された研究を活用することで、英語で発表された研究によるエビデンスが得られる地理的範囲（関連研究が存在する 2° ×2° グリッドセルの数）を（特に生物多様性の高い地域で）12～25%、また英語のエビデンスが得られる種数（関連研究の対象となっている種の数）を 5～32%拡大することができた。一方で、これら英語以外の言語で発表された研究は、より頑健でない研究デザインを利用している傾向があった。本研究の結果は、英語以外の言語で発表された研究を活用することが、地域や状況に応じたエビデンスが欠如しているという普遍的な問題を克服し、エビデンスに基づく保全を世界的に促進するための鍵となることを示している。生物多様性保全以外の地球規模の課題においても、意思決定に科学的情報を提供する上で、これまで十分に活用されてこなかった英語以外の言語による科学がどのように貢献しうるのかを厳密に再評価することが、幅広い分野で重要だと考えられる。

Translated by Tatsuya Amano
