# Supplementary material for: Tapping into non-English-language science for the conservation of global biodiversity
Source: PLoS Biol. 2021 Oct 7;19(10):e3001296. doi: 10.1371/journal.pbio.3001296 (PMC8496809; doi:10.1371/journal.pbio.3001296)
Supplement: S6 Alternative Language Abstract — (PDF) [file pbio.3001296.s019.pdf]

지구 생물다양성 보전을 위한 비영어 과학의 활용

초록

여러 학문 분야에 걸쳐 영어 이외의 언어로 쓰인 과학적 정보의 활용도가 낮은 것은 중요한 과학적 지식들이 모두 영어로 제공될 것이라는 관습적 가정에 기반한다. 그러나 비영어로 발표된 과학 연구도 대체할 수 없는 값진 과학적 정보를 제공할 수 있으며, 이는 특히 명백한 증거가 제한적이거나 현안 문제 해결을 위해 가용한 증거들을 취합하는 것이 시급한 학문분야에서 더욱 두드러진다. 그럼에도 불구하고 비영어 과학 연구들이 학계에 기여하는 바와 그 학문적 활용도는 거의 정량화되지 못한 실정이다. 이 연구는 이러한 비영어 과학 연구들이 지구 생물다양성 보전의 핵심적 근거를 제공함을 보여준다. 우리는 16 개 언어로 작성된 419,679 편의 동료 평가 논문들을 검토하여, 생물다양성 보전 활동의 효과에 대한 증거를 제공하는 1,234 개 비영어 논문 및 동일한 기준이 적용된 4,412 개의 영어 논문을 선별하였다. 관련 논문의 수가 충분히 확보된 12 개의 비영어 언어 중 6 개 언어에서는 해당 분야에 발표된 연구의 수가 증가하는 추세에 있었다. 비록 비영어 연구들은 상대적으로 덜 강력한 연구 설계를 따르는 경향이 있었지만, 영어로 된 연구들만 수행된 경우에 비해 비영어 연구들을 통합할 경우 연구의 지리적 범위(관련 연구가 수행된 2° × 2° 격자의 수)가 생물다양성이 풍부한 지역을 중심으로 12-25% 확장되었으며, 분류군의 범위(관련 연구에서 다루는 종의 수)도 5-32% 증가하는 것으로 나타났다. 이 연구 결과는 영어 이외의 언어로 쓰인 연구들을 종합하는 것이 보전을 위해 필요한 지역특이적이고 맥락의존적인 증거의 만성적 부족을 극복하고 증거에 기반한 전세계적인 보전 활동을 촉진하는 열쇠임을 보여준다. 이에 우리는 생물다양성 보전 이외의 다양한 학문 분야에서도 지구적 문제를 해결하기 위한 결정 이전에 비영어 과학 연구의 미개척 잠재성을 반드시 재고할 필요성이 있음을 강조한다.

Translated by Chang-Yong Choi and Hae-Min Seo
