# Supplementary material for: Tapping into non-English-language science for the conservation of global biodiversity
Source: PLoS Biol. 2021 Oct 7;19(10):e3001296. doi: 10.1371/journal.pbio.3001296 (PMC8496809; doi:10.1371/journal.pbio.3001296)
Supplement: S7 Alternative Language Abstract — (PDF) [file pbio.3001296.s020.pdf]

استفاده از دانش موجود به زبان های غیر انگلیسی برای حفظ تنوع زیستی جهانی

خلاصه

این فرض رایج که همه اطلاعات علمی مهم به زبان انگلیسی قابل دستیابی است، زمینه‌ساز عدم استفاده از دانش موجود به سایر زبان‌ها در رشته های مختلف شده است. این در حالیست که دانش منتشر شده به زبان‌های غیر انگلیسی اطلاعات خاص و ارزشمندی را به همراه دارد. این اطلاعات به خصوص در رشته هایی که شواهد محدود و کمیاب است و در مسائل فوری که یافتن راه حل آن‌ها نیازمند ترکیب شواهد موجود است می‌تواند بسیار ارزشمند باشد. با این وجود ، سهم دانش منتشر شده به زبان‌های غیر انگلیسی در جوامع علمی و کاربرد علم به ندرت تعیین می شود. مطالعه حاضر نشان می دهد که دانش موجود به زبان های غیر انگلیسی شواهد مهمی برای حفاظت از تنوع زیستی جهانی ارائه می دهند. با غربالگری 419،679 مقاله معتبر به 16 زبان، ما 1234 مطالعه غیر انگلیسی زبان را شناسایی کردیم که شواهدی در مورد اثربخشی مداخلات حفاظت از تنوع زیستی ارائه کرده اند. در مقایسه، تعداد مطالعات انگلیسی زبانی که با همین معیارها شناسایی شد 4412 مورد بود. تعداد مطالعات غیر انگلیسی زبان در شش زبان از 12 زبانی که تعداد کافی مطالعات مرتبط در آن ها وجود داشت با سرعت فزاینده ای در حال افزایش است. با وجود اینکه مطالعات غیر انگلیسی زبان در مجموع بر اساس طرح آزمایش‌های ضعیف تری انجام شده اند، استفاده از آن ها می تواند پوشش جغرافیایی (یعنی تعداد سلول های شبکه 2 در 2 درجه دارای مطالعات مرتبط) شواهد انگلیسی زبان را 12-25 درصد، به ویژه در مناطق با تنوع زیستی بالا، و پوشش تاکسونومیک (به عنوان مثال ، تعداد گونه های تحت پوشش مطالعات مرتبط) را 5-32 درصد افزایش دهد. نتایج ما نشان می دهد که ترکیب مطالعات غیر انگلیسی زبان برای غلبه بر فقدان گسترده شواهد محلی و موردی اهمیت زیادی داشته و می تواند نقشی کلیدی در تسهیل حفاظت مبتنی بر شواهد در سراسر جهان ایفا کند. ما جوامع علمی رشته‌های مختلف را تشویق می‌کنیم که به طور دقیق پتانسیل های بکار گرفته نشده از دانش منتشر شده به زبان های غیر انگلیسی را در تصمیمات مربوط به حل چالش های جهانی مورد ارزیابی مجدد قرار دهند.

Translation by: Elham Nourani
