# Supplementary material for: Tapping into non-English-language science for the conservation of global biodiversity
Source: PLoS Biol. 2021 Oct 7;19(10):e3001296. doi: 10.1371/journal.pbio.3001296 (PMC8496809; doi:10.1371/journal.pbio.3001296)
Supplement: S8 Alternative Language Abstract — (PDF) [file pbio.3001296.s021.pdf]

Wykorzystanie nieanglojęzycznych źródeł naukowych w globalnej ochronie bioróżnorodności

Abstrakt

Powszechnie przyjęte założenie, że każda ważna informacja naukowa jest dostępna w języku angielskim, przyczynia się do niedostatecznego wykorzystania wiedzy dostępnej w innych językach. Można jednak przypuszczać, że nieanglojęzyczne źródła naukowe przynoszą unikalne i cenne informacje, zwłaszcza w dyscyplinach, w których wiedza jest niepełna, oraz dla nowych problemów, gdzie synteza dostępnych faktów jest pilnym wyzwaniem. Jednak taki wkład wiedzy niedostępnej w języku angielskim i zastosowanie nauki rzadko jest określone ilościowo. W tym opracowaniu dowodzimy, że badania opublikowane w lokalnych językach dostarczają podstawowej wiedzy dotyczącej ochrony bioróżnorodności na świecie. Spośród przeanalizowanych 419 679 recenzowanych artykułów naukowych w 16 językach, zidentyfikowaliśmy 1234 nieanglojęzyczne publikacje dostarczające dowody na skuteczność interwencji na rzecz ochrony bioróżnorodności, w porównaniu z 4412 anglojęzycznymi badaniami, które spełniały te same kryteria. Takie nieanglojęzyczne prace są publikowane coraz częściej w sześciu z 12 języków, w których była wystarczająca liczba spełniających kryteria badań. Uwzględnienie badań nieanglojęzycznych może wzbogacić informacje anglojęzyczne poprzez rozszerzenie zasięgu geograficznego (tj. liczby komórek siatki 2° × 2° wraz z odpowiednimi badaniami) o 12-25%, zwłaszcza w regionach różnorodnych biotycznie, oraz zasięgu taksonomicznego (tj. liczby gatunków objętych odpowiednimi badaniami) o 5-32%, pomimo, że zazwyczaj opierają się one na mniej solidnie zaplanowanych badaniach. Nasze wyniki dowodzą, że synteza badań nieanglojęzycznych jest kluczem do przezwyciężenia powszechnego braku lokalnych, zależnych od kontekstu wyników i ułatwienia ochrony przyrody opartej na danych z całego świata. Postulujemy, aby wykorzystywać potencjał naukowych źródeł nieanglojęzycznych do nowej i rygorystycznej oceny w podejmowaniu decyzji dotyczących innych niż ochrona bioróżnorodności globalnych wyzwań.

Translated by Joanna Kajzer-Bonk
