# Supplementary material for: Tapping into non-English-language science for the conservation of global biodiversity
Source: PLoS Biol. 2021 Oct 7;19(10):e3001296. doi: 10.1371/journal.pbio.3001296 (PMC8496809; doi:10.1371/journal.pbio.3001296)
Supplement: S9 Alternative Language Abstract — (PDF) [file pbio.3001296.s022.pdf]

Aproveitando a ciência em língua não inglesa para a conservação da biodiversidade global

A suposição amplamente aceita de que qualquer informação científica importante estaria disponível em inglês fundamenta a subutilização da ciência publicada em outros idiomas em todas as disciplinas. Ao mesmo tempo, espera-se que a ciência publicada em outros idiomas traga informações científicas únicas e valiosas, especialmente em disciplinas onde as evidências são esparsas, e para questões emergentes em que sintetizar as evidências disponíveis é um desafio urgente. Porém, essa contribuição da ciência publicada em língua não inglesa para as comunidades científicas e a aplicação da ciência raramente é quantificada. Neste trabalho, nós mostramos que estudos publicados em idiomas diferentes do inglês fornecem evidências cruciais para informar ações de conservação da biodiversidade global. Ao examinar 419.679 artigos revisados por pares e escritos em 16 idiomas, nós identificamos 1.234 estudos em idiomas diferentes do inglês que fornecem evidências sobre a eficácia de ações de conservação da biodiversidade, em comparação com 4.412 estudos escritos em inglês que atendiam com os mesmos critérios. Estudos relevantes sobre a eficácia de ações de conservação da biodiversidade em idiomas diferentes do inglês estão sendo publicados a uma taxa crescente em seis dos 12 idiomas onde havia um número suficiente de estudos relevantes. A incorporação de estudos em idiomas diferentes do inglês pode expandir a cobertura geográfica (isto é, o número de células 2° × 2° com estudos relevantes) de evidências em língua inglesa em 12 a 25%, especialmente em regiões biodiversas, e a cobertura taxonômica (isto é, o número de espécies abordadas nos estudos relevantes) em 5 a 32%, embora estudos em idiomas diferentes do inglês tendam a se basear em desenhos amostrais menos robustos. Nossos resultados mostram que sintetizar estudos em idiomas diferentes do inglês é fundamental para superar a falta generalizada de evidências locais e dependentes do contexto e facilitar globalmente a conservação baseada em evidências. Nós incentivamos as diversas disciplinas a reavaliar rigorosamente o potencial inexplorado da ciência publicada em idiomas diferentes do inglês para informar as decisões de enfrentamento a outros desafios globais.

Translated by Rafael D. Zenni
