# Supplementary material for: Tapping into non-English-language science for the conservation of global biodiversity
Source: PLoS Biol. 2021 Oct 7;19(10):e3001296. doi: 10.1371/journal.pbio.3001296 (PMC8496809; doi:10.1371/journal.pbio.3001296)
Supplement: S10 Alternative Language Abstract — (PDF) [file pbio.3001296.s023.pdf]

Includerea studiilor științifice publicate în alte limbi decât engleză pentru a conserva biodiversitatea globală

Rezumat

Ipoteza larg acceptată că orice informație științifică importantă este disponibilă în limba engleză stă la baza utilizării foarte limitate a studiilor științifice nepublicate în limba engleză, iar această tendintă e valabilă în practic toate disciplinele. Cu toate acestea, se așteaptă ca lucrările științifice publicate in limbi diferite de engleză să conţină informații științifice unice și valoroase, în special în disciplinele în care dovezile disponibile sunt incomplete și pentru problemele emergente, în cazul cărora sinteza informaţiilor reprezintă o provocare urgentă. O astfel de contribuție a literaturii științifice publicate în alte limbi decât engleza pentru comunitățile științifice și în implementarea practică a științei este însă rareori cuantificată în domeniul conservării biodiversității la nivel mondial. Aici demonstrăm că studiile publicate în alte limbi decât engleză oferă dovezi cruciale pentru informarea cu privire la conservarea biodiversității la nivel global. Prin examinarea a 419 679 de studii științifice evaluate *inter pares* (peer-reviewed) si publicate în 16 limbi, am identificat 1 234 de studii publicate în alte limbi decât engleză şi care au furnizat dovezi cu privire la eficacitatea intervențiilor de conservare a biodiversității, comparativ cu 4 412 studii publicate în limba engleză, identificate cu aceleași criterii. Studiile relevante, altele decât cele în limba engleză, sunt publicate într-un ritm accelerat în șase din cele 12 limbi în care a existat un număr suficient de studii relevante. Încorporarea studiilor care nu sunt în limba engleză poate extinde acoperirea geografică (adică numărul de 2° × 2° celule de rețea cu studii relevante) al dovezilor disponibile în limba engleză cu 12-25%, în special în regiunile cu biodiversitate ridicată, și acoperirea taxonomică (adică numărul de specii care fac obiectul studiilor relevante) cu 5-32%, deși aceste studii tind să se bazeze pe metode de studiu mai puțin robuste. Rezultatele noastre arată că sinteza studiilor publicate în alte limbi decât engleză este esențială pentru depășirea lipsei pe scară largă de dovezi locale, dependente de context și pentru facilitarea conservării biodiversităţii bazate pe dovezi la nivel global. Îndemnăm şi alte discipline să reevalueze riguros potențialul neexploatat pe care studiile științifice nepublicate în engleză îl reprezintă pentru luarea în cunoştiinţă de cauză a deciziilor de abordare a altor provocări globale.

Translated by Silviu Petrovan
