# Supplementary material for: Tapping into non-English-language science for the conservation of global biodiversity
Source: PLoS Biol. 2021 Oct 7;19(10):e3001296. doi: 10.1371/journal.pbio.3001296 (PMC8496809; doi:10.1371/journal.pbio.3001296)
Supplement: S11 Alternative Language Abstract — (PDF) [file pbio.3001296.s024.pdf]

Использование неанглоязычных научных данных для охраны глобального биоразнообразия

Аннотация

Широко распространенное мнение, что любая важная научная информация доступна на английском языке, приводит к недостаточному использованию неанглоязычных данных в разных областях науки. В то же время, неанглоязычная информация может быть уникальной и важной, особенно в областях, где свидетельства разрозненны и где обобщение доступных свидетельств необходимо для срочного решения проблем. Несмотря на это, количественная оценка вклада неанглоязычной информации в науку и ее практическое применение проводится редко. Наше исследование показало, что неанглоязычные исследования содержат ключевые данные для информирования охраны биоразнообразия в мире. Мы проанализировали содержание 419,679 рецензируемых публикаций на 16 языках и выявили 1,234 неанглоязычные публикации об эффективности природоохранных мероприятий по сравнению с 4,412 англоязычными публикациями, отобранными по тем же критериям. На шести языках из 12, по которым было достаточное количество исследований, научная литература публикуется особенно активно. Включение неанглоязычных исследований может расширить географический охват (т.е. количество 2° × 2° ячеек) англоязычных исследований на 12-25%, особенно в богатых биоразнообразием регионах, а таксономический охват (т.е. количество изученных видов) на 5-32%, несмотря на то, что неанглоязычные исследования имеют тенденцию к использованию менее надежных методик. Данная работа показывает, что использование неанглоязычных научных данных является ключевым фактором для преодоления разрозненности природоохранных работ и усовершенствования охраны природы по всему миру на основе научно обоснованных свидетельств. Мы призываем к серьезному пересмотру неиспользованного потенциала неанглоязычной научной литературы в разных областях для информирования решений, направленных на преодоление других глобальных проблем.

Translated by I. Khorozyan
