# Supplementary material for: Tapping into non-English-language science for the conservation of global biodiversity
Source: PLoS Biol. 2021 Oct 7;19(10):e3001296. doi: 10.1371/journal.pbio.3001296 (PMC8496809; doi:10.1371/journal.pbio.3001296)
Supplement: S12 Alternative Language Abstract — (PDF) [file pbio.3001296.s025.pdf]

非英语研究对于保护全球生物多样性的潜力

摘要

人们普遍接受的前提：任何重要的科学信息都应以英语来呈现。这使得很多学科忽视了非英语发表的科学研究。这类研究可以带来独特的、有价值的科学信息；特别针对那些证据不完整的学科；或是在应对紧迫议题的挑战而需要对现有学术资源进行整合的时候。尽管如此，很少有人去评估非英语研究究竟对科研界和科学成果应用方面所产生的贡献。在这项研究中，我们证明了非英语研究为全球生物多样性保护提供了重要证据。在以 16 种语言发表的 419,679 篇同行评议论文中，我们最终筛选出 1,234 项非英语研究进行分析。我们的结果显示：这些研究为生物多样性保护干预措施的有效性提供了重要的证据。我们既而采用同样的标准对 4,412 项英语研究进行对比。在样本充足且相关性高的 12 种非英语研究中，6 种语言的研究正在以更快的速率发表。使用非英语研究可以把英语研究发表的证据所覆盖地理范围（即具有相关研究的 2° × 2° 栅格单元的数量）扩大 12-25%，尤其是在生物多样性较高的区域；非英语研究把所涉及的物种多样性（即相关研究涵盖的物种数量）增加了 5-32%，尽管有时一些研究可能基于不太稳健的实验设计。综上，我们的研究提示：综合非英语研究是解决全球范围内普遍缺乏与环境相关的在地证据，并促进全球范围内基于实证的保护工作的关键。为此，我们敦促生物多样性保护之外的其他学科应严格评估非英语研究所提供的信息，特别是在为解决其他全球挑战提供决策方面所蕴藏的巨大潜力。

Translated by Yang Liu
