# Supplementary material for: Tapping into non-English-language science for the conservation of global biodiversity
Source: PLoS Biol. 2021 Oct 7;19(10):e3001296. doi: 10.1371/journal.pbio.3001296 (PMC8496809; doi:10.1371/journal.pbio.3001296)
Supplement: S13 Alternative Language Abstract — (PDF) [file pbio.3001296.s026.pdf]

Aprovechar la ciencia en idiomas distintos al inglés para la conservación global de la biodiversidad.

Resumen

La suposición ampliamente aceptada de que cualquier información científica se encontraría disponible en el inglés cimienta la subutilización de la ciencia disponible en otros idiomas en todas las disciplinas. Al mismo tiempo, se espera que la ciencia publicada en otros idiomas distintos al inglés, aporten información científica única y valiosa, especialmente en disciplinas en las que la evidencia es escasa, y para temas emergentes, en los que la síntesis de la evidencia disponible es un reto urgente. Sin embargo, la contribución de la ciencia publicada en idiomas distintos al inglés a las comunidades científicas y a la aplicación de la ciencia rara vez se cuantifica. En este artículo, mostramos que los estudios publicados en idiomas distintos del inglés aportan evidencia científica crucial para informar las acciones de conservación de la biodiversidad a nivel global. Al examinar 419.679 artículos revisados por pares escritos en 16 idiomas, identificamos 1.234 estudios en idiomas distintos del inglés que aportan evidencia sobre la eficacia de las acciones de conservación de la biodiversidad, en comparación con 4.412 estudios escritos en inglés que cumplían los mismos criterios. Los estudios relevantes sobre la eficacia de las acciones de conservación de la biodiversidad en idiomas distintos del inglés se han publicado a un ritmo creciente en seis de los 12 idiomas en los que ha habido un número suficiente de estudios relevantes. La incorporación de estudios en idiomas distintos del inglés puede ampliar la cobertura geográfica de la evidencia en inglés entre un 12 y un 25% (es decir, el número de celdas de 2° × 2° con estudios pertinentes), especialmente en las regiones biodiversas, y puede también ampliar la cobertura taxonómica (es decir, el número de especies abordadas en los estudios relevantes) entre un 5 y un 32%. Sin embargo, los estudios en idiomas distintos del inglés suelen basarse en diseños de muestreo menos robustos. Nuestros resultados muestran que incluir síntesis de estudios en idiomas distintos del inglés es fundamental para superar la falta de evidencia local y que depende del contexto, facilitando la conservación basada en evidencia a nivel global. Animamos a otras disciplinas a reevaluar con rigor el potencial no aprovechado de la ciencia publicada en idiomas distintos del inglés para fundamentar las decisiones que se tomen para afrontar otros retos globales.

Translated by Violeta Berdejo-Espinola
