# Supplementary material for: Tapping into non-English-language science for the conservation of global biodiversity
Source: PLoS Biol. 2021 Oct 7;19(10):e3001296. doi: 10.1371/journal.pbio.3001296 (PMC8496809; doi:10.1371/journal.pbio.3001296)
Supplement: S14 Alternative Language Abstract — (PDF) [file pbio.3001296.s027.pdf]

非英文研究對全球生物多樣性保育的貢獻

摘要

一般普遍認為，透過英文就能獲得任何重要的科學知識，這樣的誤解是基於我們尚未充分瞭解各學科領域的非英文研究。非英文論文帶有獨一無二且值得深究的科學知識，尤其是科學證據不完備的學科領域，以及急需綜整科學證據的議題，都是迫切的挑戰。但是，非英文研究對科學知識及應用的貢獻卻極少量化探討。在此，我們呈現了非英文研究為全球生物多樣性提供諸多保育證據。經過篩選遍及 16 種語言、經同儕審查的 419,679 篇非英文研究，發現其中 1,234 篇為生物多樣性保育行動提供有效證據，並與 4,412 篇英文研究以相同標準比較。在論文篇數充足的 12 種非英文種語言中，6 種語言的論文發表量正在快速成長。儘管有些非英文研究的試驗設計基礎確實不算嚴謹，然而，納入非英文研究，能將保育證據的空間涵蓋範圍（研究地點所在的經度 2° × 緯度 2° 的網格數量）擴大 12-25%，尤其集中在生物多樣性熱點。同時可將分類群涵蓋範圍（研究對象所屬生物類群所涉及的物種數）提高 5-32%。研究結果顯示，綜整非英文研究是克服缺乏在地背景脈絡相關的保育證據，以及增加全球保育證據的關鍵。我們鼓勵更多學科領域重新審慎評估非英文研究在面對其他全球挑戰所需的決策資訊中，尚未探索的潛在價值。

Translator: Da-Li Lin
