# Supplementary material for: Tapping into non-English-language science for the conservation of global biodiversity
Source: PLoS Biol. 2021 Oct 7;19(10):e3001296. doi: 10.1371/journal.pbio.3001296 (PMC8496809; doi:10.1371/journal.pbio.3001296)
Supplement: S15 Alternative Language Abstract — (PDF) [file pbio.3001296.s028.pdf]

Küresel biyoçeşitliliğin korunması için İngilizce dışındaki dillerde yayınlanan çalışmalardan faydalanılması

Öz

Herhangi bir önemli bilimsel bilginin İngilizce olarak bulunabileceğine dair yaygın olarak kabul edilen varsayım, İngilizce dilinde yayınlanmayan disiplinler arasında bilimsel çalışmaların yetersiz kullanımının temelini oluşturmaktadır. Bununla birlikte, özellikle bulguların düzensiz olduğu ve ortaya çıkan acil durumlar için mevcut bulguların sentezlenmesinin önemli bir zorluk olduğu durumlar için İngilizce olmayan dillerdeki bilimsel çalışmaların özgün ve değerli bilimsel bilgiler getirmesi beklenmektedir. Yine de İngilizce olmayan bilimsel çalışmaların bilimsel topluluklara ve bilimsel uygulamalara katkısı nadiren ölçülmektedir. Bu çalışmada İngilizce olmayan araştırmaların, küresel biyoçeşitliliğin korunmasında çok önemli kanıtlar sağladığını gösteriyoruz. 16 dilde 419.679 hakemli makale taranarak, aynı kriterlerle tanımlanan 4.412 İngilizce yayınlanan çalışmaya kıyasla, biyolojik çeşitliliği koruma müdahalelerinin etkinliğine dair kanıt sağlayan İngilizce olmayan 1.234 çalışmayı belirledik. Yeterli sayıda ilgili çalışmanın bulunduğu 12 dilden altısında İngilizce olmayan ilgili çalışmalar artan bir oranda yayınlanmaktadır. İngilizce olmayan çalışmaların güvenirliliğinin daha az olmasına rağmen, bu çalışmaların dahil edilmesi İngilizce dilinde yayınlanan bulguların coğrafi kapsamını (örn., ilgili çalışmalarla birlikte 2° × 2° ızgara hücrelerinin sayısı) %12-25 oranında, özellikle biyolojik çeşitliliği yüksek olan bölgelerde taksonomik kapsamı (örn., ilgili çalışmaların kapsadığı türlerin sayısı) %5-32 oranında artırmaktadır. Sonuçlarımız, İngilizce olmayan çalışmaların sentezlenmesinin, yaygın yerel, bağlama bağlı kanıt eksikliğinin üstesinden gelmenin ve küresel boyutta bulgulara dayalı korumayı kolaylaştırmanın anahtarı olduğunu göstermektedir. Diğer disiplinleri de başka küresel zorlukları ele almak amacıyla, önemli kararların verilmesinde İngilizce olmayan bilimin kullanılmayan potansiyelini titizlikle yeniden değerlendirmeye çağırıyoruz.

Translated by M. Çisel Kemahlı Aytekin
