# Supplementary material for: Tapping into non-English-language science for the conservation of global biodiversity
Source: PLoS Biol. 2021 Oct 7;19(10):e3001296. doi: 10.1371/journal.pbio.3001296 (PMC8496809; doi:10.1371/journal.pbio.3001296)
Supplement: S16 Alternative Language Abstract — (PDF) [file pbio.3001296.s029.pdf]

Використання неангломовних наукових досліджень для збереження глобального біорізноманіття

Резюме

Широко поширене припущення, що будь-яка важлива наукова інформація доступна англійською мовою, лежить в основі недостатньої уваги до неангломовних досліджень. Однак, ці дослідження можуть містити унікальну та цінну наукову інформацію, особливо відносно дисциплін, де наукові свідчення представлені нерівномірно, та питань, що нагально потребують узагальнення наявних знань. Наразі внесок неангломовних досліджень для наукових спільнот та застосувань науки оцінюється рідко. Ми ж показуємо, що неангломовні дослідження є важливими джерелом інформації щодо збереження біорізноманіття у всьому світі. Опрацювавши 419679 рецензованих статей, опублікованих 16 мовами, ми виявили 1234 неангломовних дослідження, які містять інформацію про ефективність природоохоронних заходів, порівняно з 4412 англомовними дослідженнями, визначеними за тими ж критеріями. Серед шести з 12 неанглійських мов, які були достатньо представлені, кількість опублікованих досліджень пришвидшується. Неангломовні дослідження дозволяють розширити географічне охоплення (число квадратів 2° × 2° сітки) англомовної інформації на 12-25%, особливо в регіонах з високим біорізноманіттям, та таксономічне охоплення (число видів) на 5-32%. Водночас, дані з неангломовних джерел, як правило, базуються на менш надійних дослідженнях. Наші результати показують, що узагальнення неангломовних досліджень є надзвичайно важливим для подолання поширеної нестачі локальних, залежних від контексту наукових свідчень та забезпечення доказової природоохоронної діяльності. Ми закликаємо ширші наукові кола ретельно переосмислити невикористаний потенціал неангломовних досліджень для обґрунтування рішень щодо вирішення різноманітних глобальних проблем.

Переклад: Марина Голівець (Translated by: Marina Golivets)
